# Complications of Absorbable Fixation in Maxillofacial Surgery: A Meta-Analysis

**DOI:** 10.1371/journal.pone.0067449

**Published:** 2013-06-28

**Authors:** Liya Yang, Meibang Xu, Xiaolei Jin, Jiajie Xu, Jianjian Lu, Chao Zhang, Tian Tian, Li Teng

**Affiliations:** Department 2 of Cranio-maxillo-facial Surgery, Plastic Surgery Hospital, Chinese Academy of Medical Sciences and Peking Union Medical College, Beijing, P.R. China; Harvard Medical School, United States of America

## Abstract

**Background:**

The use of titanium during maxillofacial fixation is limited due to its palpability, mutagenic effects and interference with imaging, which lead to the requirement for subsequent removal. The use of a biologically absorbable fixation material will potentially eliminate these limitations. In this meta-analysis, we analyzed the complications of absorbable fixation in maxillofacial surgery.

**Methods:**

We performed a systematic search of PubMed, Embase, Cochrane Central Register of Systematic Reviews and Cochrane Central Register of Controlled Trials for trials published through December 2012. Data extracted from literature were analyzed with Review manager 5.0.24.

**Results:**

Relevant data was extracted from 20 studies (1673 participants) and revealed that patients in the absorbable group had significantly more complications than those in the titanium group (RR = 1.20; 95% CI: 1.02–1.42; P = 0.03) in all enrolled maxillofacial surgeries. For bimaxillary operation subgroup, the absorbable fixation group did not have a significant increase in complications when compared with the titanium group (RR = 1.89; 95% CI: 0.85–4.22; P = 0.12). There was no significant difference observed between the absorbable and titanium groups receiving a bilateral sagittal split ramus osteotomy (BSSRO) (RR = 1.45; 95% CI: 0.84–2.48; P = 0.18) and Le Fort I osteotomy (RR = 0.65; 95% CI: 0.34–1.23; P = 0.18). The combined results of the five trials revealed that the absorbable group had a significantly lower rate of complications compared to the titanium group (RR = 0.71; 95% CI: 0.52–0.97; P = 0.03) in fracture fixation.

**Conclusion:**

This meta-analysis shows that absorbable fixation systems used for fixation in maxillofacial surgery do not have adequate safety profiles. Subgroup indicated the safety of absorbable fixation systems was superior during fracture fixation. The absorbable fixation systems tend to have a similar favorable safety profile as titanium fixation during Le Fort I, bimaxillary operation and BSSRO.

## Introduction

Essential prerequisites for bone healing of fractures and osteotomies include sufficient vascularization, immobilization of bone segments and anatomical reduction. Previously the only method of achieving this was by intraosseous wiring coupled with rigid intermaxillary (upper to lower jaw) fixation. Recent developments in biomaterials have led to the achievement of fixation using titanium. This allows patients to functionally load their masticatory system immediately following surgery [1]. However, as the need for fixation is only temporary and metallic materials cause stress shielding of the underlying bone, the removal of these plates after the bone has healed has been suggested [2]. The titanium implants are removed following bone healing in a second operation in 5–40% of the cases [3]. Moreover, titanium particles have been found in scar tissue covering these plates as well as in locoregional lymph nodes and an imperfect contact will occur between the metal plate and bone surface. Recently, it was reported that titanium miniplates is a new risk factor for the development of the bisphosphonate-related osteonecrosis of the jaw [4].

The use of the biologically inert and resorbable plates will potentially eliminate these limitations of titanium fixation, which may offer some clinical advantages for the fixation of facial bones during orthognathic surgery. Studies have demonstrated that maxillary stability can be achieved with satisfactory results when u-hydroxyapatite/poly-(L-lactic) acid (u-HA/PLLA) and poly-L-lactic acid (PLLA) plates are used, similar to titanium plates [5]. The resorbable system is a good system for rigid internal fixation in specific conditions where muscular and stress forces are not a determining factor in fragment displacement [6]. However, concerns remain about the stability of fixation, the length of time required for their degradation and especially the possibility of complications, such as foreign body reactions. Park et al. suggested that resorbable plate and screw systems (RPSSs) should be selected carefully depending on the fracture site and whether there is an accompanying infection. It is important to select the method that best fits the patient’s situation [7]. The use of biodegradable plates should be recommended for minimally loaded situations [8]. In addition, the process of degradation of these devices into carbon dioxide and water may take as long as 2 years [9]. Therefore, the use of resorbable plates and screws remains unpopular for internal fixation among oral and maxillofacial surgeons. Although a number of clinical studies regarding the safety of absorbable materials in maxillofacial fixation have been recently published, there is no systemic review to analyze the exact safety of absorbable materials in maxillofacial surgery. Therefore, we performed a meta-analysis to assess the safety of absorbable materials versus metal treatments (titanium) in patients receiving maxillofacial surgery.

## Materials and Methods

### Data Collection

The aim of this meta-analysis was to include all publicly available data on the treatment of maxillofacial fixation with an absorbable plate and/or screws from comparative studies or randomized controlled trials (RCTs). Two authors performed systematic searches of the medical literature to identify articles from PubMed, Embase, Cochrane Central Register of Systemic Reviews and Cochrane Central Register of Controlled Trials according to a standardized protocol, to December 2012. We conducted a comprehensive literature search with the following medical subject headings: bicortical resorbable, Poly-L-Lactic Acid, PLLA, PLLA-PGA, resorbable, bio-resorbale, biodegradable and titanium, nonresorbable, and metal. For the plates and/or screws, we also performed searches for each type separately such as screw, plate, miniplate and miniscrew. The search was limited to the English language and studies conducted in humans.

### Study Selection

Paired reviewers (L.-Y.Y. and M.-B.X.) independently evaluated references for eligibility using a two-stage procedure. In the first stage, all identified abstracts were evaluated for appropriateness to the study aim. All potentially relevant trials were retrieved and selected for full-text review to determine whether or not they met all eligibility criteria in the second stage. Articles that were selected by either reviewer were assessed, and the inclusion and exclusion criteria were evaluated by both reviewers in the second stage. Any disagreements were resolved by discussion. Eligibility criteria for the studies included the following: (1) Type of participants: patients who had received maxillofacial surgery, including bilateral sagittal split ramus osteotomy (BSSRO), intraoral vertical ramus osteotomy (IVRO), Le Fort I osteotomy or maxillofacial fracture fixation; (2) Intervention: fixed by plates and/or screws; (3) outcome measures: complications. After extraction, four categories (complications in bimaxillary operation, bilateral sagittal split ramus osteotomy (BSSRO), Le Fort I, and fracture fixation) were of the most interest to us; (4) Type of publication: only full papers on original patient data reporting absorbable treatment were considered for further analysis; and (5) Type of study: studies had to be compared studies or RCTs comparing absorbable with non-absorbable (titanium) plates and/or screws. Exclusion criteria were the use of an alveolar bone implant, maxillofacial model, cadaver, or animals.

### Data Extraction and Quality Assessment

Data concerning the type and number of interferences were extracted and entered onto specially developed forms by two reviewers, and then the verified data were entered into a Microsoft Excel spreadsheet (XP professional edition; Microsoft Corp, Redmond, WA, USA). Trial characteristics, including the disease, mean age of included patients, operation, follow-up period, and absorbable and non-absorbable materials, were collected in detail. Unpublished data were not included. We assessed the methodological quality using the Jadad score, which assigns points (maximum of 7 points) for the following parameters: randomization (2 points), method of randomization generation (2 points), double blinding (2 points) and loss to follow-up (1 point) [10].

### Data Synthesis and Analysis

All statistical analyses were performed using Review Manager 5.0.24 statistical software (Cochrane Collaboration, Oxford, United Kingdom) for the meta-analysis. As dichotomous outcomes, the 95% confidence intervals (CI) at the end of treatment were calculated for individual trials, and the relative risk (RR) was used as a summary estimator. The fixed-effect model weighted by the Mantel-Haenszel method was used, and the random effect model was used in the case of significant heterogeneity (P value of x^2^ test <0.05 and I^2^>50%). A funnel plot test was used to assess for evidence of publication bias. Forest plots were used for graphic representation of data. The surface area of the blue square represents the relative quantitative contribution of the trial to the analysis (weight) and the horizontal line indicates the 95% CI. The diamond-shaped symbol is the summary estimate of effect expressed as a RR with 95% CIs, which is an average of the pooled treatment effects across all trials. A P<0.05 was considered statistically significant.

## Results

### Study Identification

**Figure 1:**
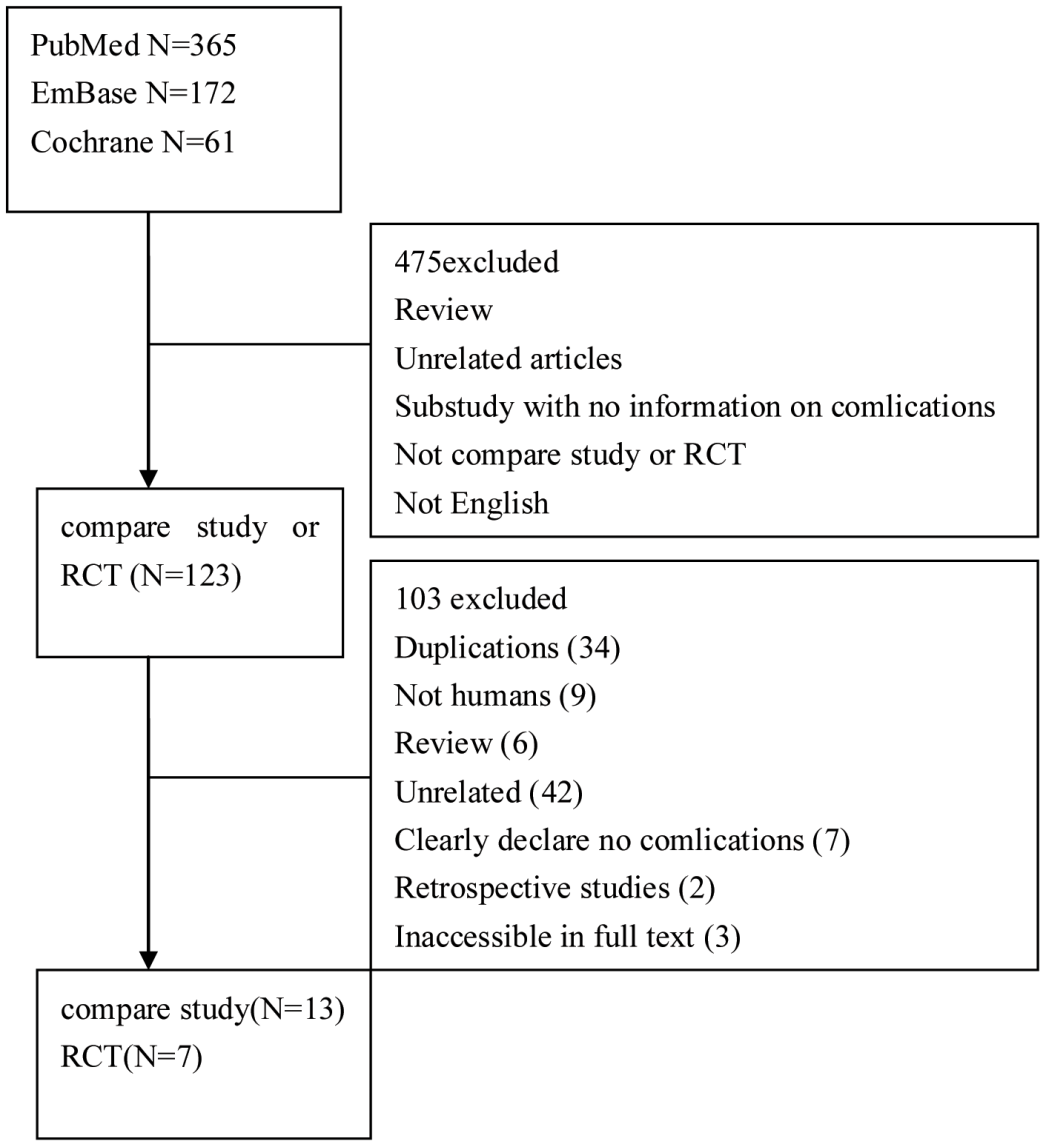
Study flow diagram.

The process of identifying eligible studies is summarized in Figure 1. After title and abstract evaluation, 123 articles were identified for further assessment. After a full-text review, 27 met the criteria for inclusion. Complications were reported in 20 of the 27 studies and included 7 RCTs and 13 comparative studies, published between 2002 and 2012.

The characteristics of the included studies are summarized in the Table S1. The 20 studies enrolled a total of 1673 participants (898 in the absorbable group and 775 in the non-absorbable group). According to the operations, three studies described multiple operations termed bimaxillary operation [11], [12], [13], which consisted of SSRO plus Le Fort I. Six studies were related to BSSRO [14], [15], [16], [17], [8], [18]. The Le Fort I subgroup included two studies [19], [20] and five studies belonged to the fracture fixation subgroup [21], [22], [23], [24], [25]. The remaining four studies can’t be classified as they included different kinds of maxillofacial surgeries [26], [27], [28], [29]. The absorbable materials were referred to poly(L-lactide-co-D/L-lactide (P (L/DL) LA), poly(L-lactic-co-glycolic acid) (PLGA), polyglycolic acid (PGA), PLLA, LactoSorb, Delta and INION, while the non-absorbable materials were titanium. The number of participants in each study ranged from 10 to 210 individuals and the age of individuals ranged from 11 to 71 y.

### Absorbable Versus Non-absorbable Group: Complication in All Enrolled Studies

**Figure 2:**
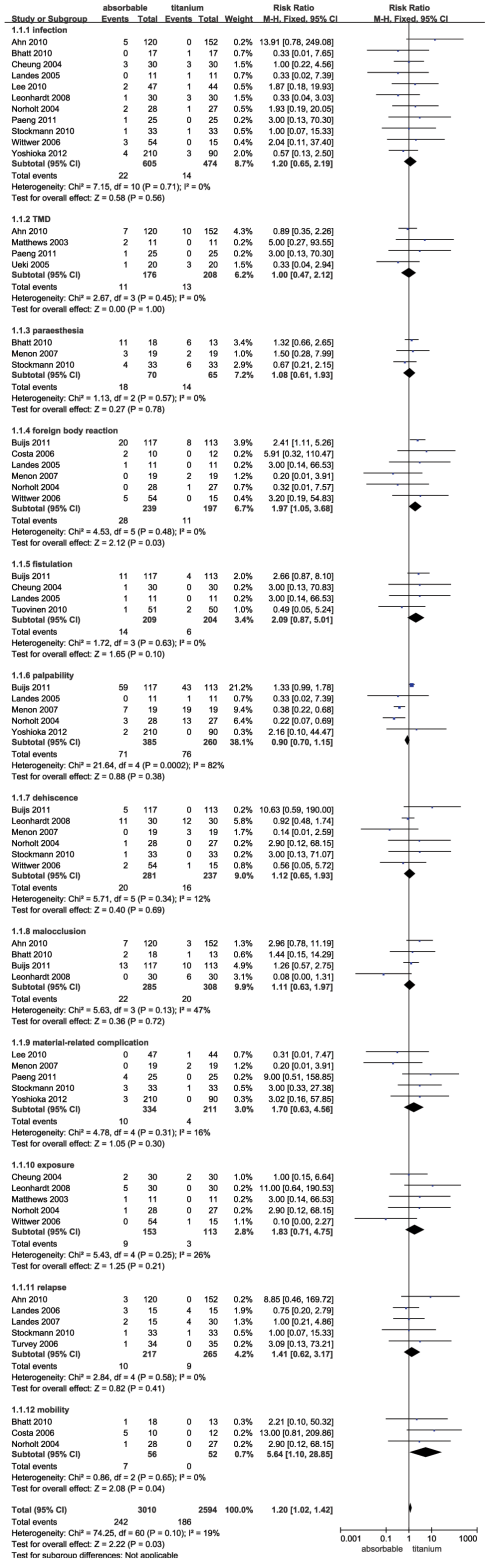
Forest plot of trials of absorbable fixation versus titanium examining the effect on relative risk of complications in all enrolled trials. TMD = temporomandibular joint dysfunction; df = degrees of freedom; M-H = Mantel-Haenszel.

**Figure 3:**
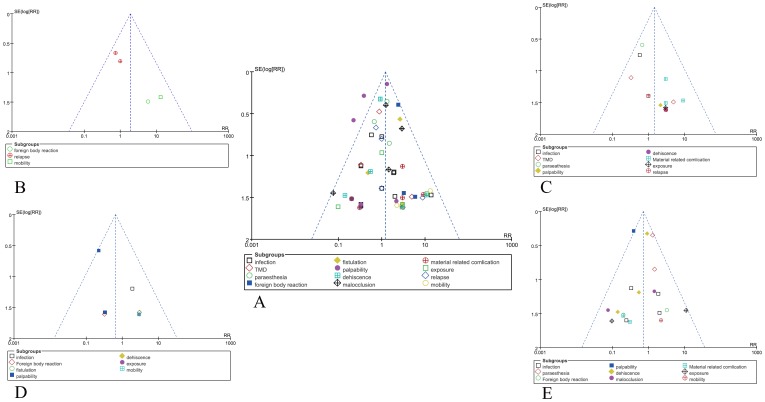
Funnel plot to assess for evidence of publication bias. 3a. Funnel plot for the complication in all studies; 3b. Funnel plot for the complication in bimaxillary operations; 3c. Funnel plot for the complication in BSSRO; 3d. Funnel plot for the complication in Le Fort I; 3e. Funnel plot for the complication in fracture fixation.

The combined results of the 20 trials revealed that the absorbable group had significantly more complications when compared with the titanium group (RR = 1.20; 95% CI: 1.02–1.42; P = 0.03). The heterogeneity test was not substantial, as assessed by the I^2^ statistics (Q [d.f. = 60] = 74.25; P = 0.10; I^2^ = 19%) (Figure 2). A sub-group analysis was performed for complications, including infection, temporomandibular joint dysfunction (TMD), paraesthesia, foreign body reaction (local inflammation and redness), fistulation, palpability, dehiscence, malocclusion, material-related complication (loose screw, screw head fracture and plate fracture), exposure, relapse and mobility. Foreign body reaction (RR = 1.97; 95% CI: 1.05–3.68; P = 0.03) and mobility (RR = 5.64; 95% CI: 1.10–28.85; P = 0.04) occurred significantly more frequently in patients receiving absorbable fixation compared to patients receiving non-absorbable fixation. However, the absorbable group was not associated with a more significant increase in infection (RR = 1.20; 95% CI: 0.65–2.19; P = 0.56), TMD (RR = 1.00; 95% CI: 0.47–2.12; P = 1.00), paraesthesia (RR = 1.08; 95% CI: 0.61–1.93; P = 0.78), fistulation (RR = 2.09; 95% CI: 0.87–5.01; P = 0.10), palpability (RR = 0.90; 95% CI: 0.70–1.15; P = 0.38), dehiscence (RR = 1.12; 95% CI: 0.65–1.93; P = 0.69), malocclusion (RR = 1.11; 95% CI: 0.63–1.97; P = 0.72), material related complication (RR = 1.70; 95% CI: 0.63–4.56; P = 0.30), exposure (RR = 1.83; 95% CI: 0.71–4.75; P = 0.21) and relapse (RR = 1.41; 95% CI: 0.62–3.17; P = 0.41). Publication bias was not evident, as estimated by the funnel plot for the studies on complications (Figure 3a).

### Complication Comparison in the Bimaxillary Operation

**Figure 4:**
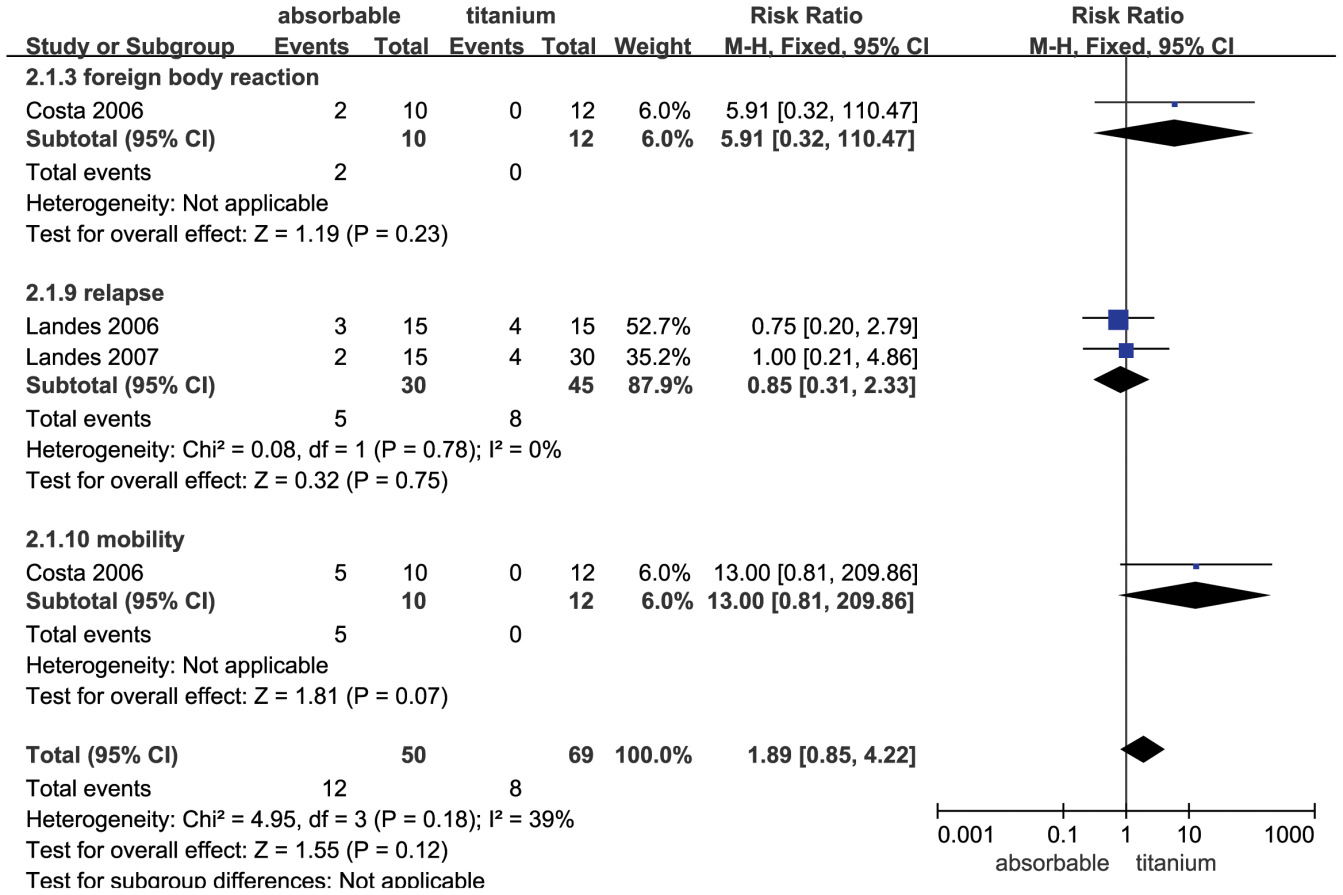
Forest plot of trials of absorbable fixation versus titanium examining the effect on relative risk of complications in bimaxillary operation. See Figure 2 legend for expansion of abbreviations.

There were 3 trials comparing the complications of absorbable and titanium fixation in the bimaxillary operation category (BSSRO plus Le Fort I). We found that the application of absorbable fixation did not have a significant increase in complications compared with titanium (RR = 1.89; 95% CI: 0.85–4.22; P = 0.12). The heterogeneity was not evident (Q [d.f. = 3] = 4.95; P = 0.18, I^2^ = 39%) (Figure 4). A sub-group analysis of complications was also performed that included foreign body reaction, relapse and mobility. The group receiving absorbable fixation was not associated with a more significant increase in foreign body reaction (RR = 5.91; 95% CI: 0.32–110.47; P = 0.23), relapse (RR = 0.85; 95% CI: 0.31–2.33; P = 0.75) and mobility (RR = 13.00; 95% CI: 0.81–209.86; P = 0.07). Funnel plot for the studies on complications in the bimaxillary operation category was relatively symmetrical, and publication bias was not evident (Figure 3b).

### Complication Comparison in the BSSRO Operation

**Figure 5:**
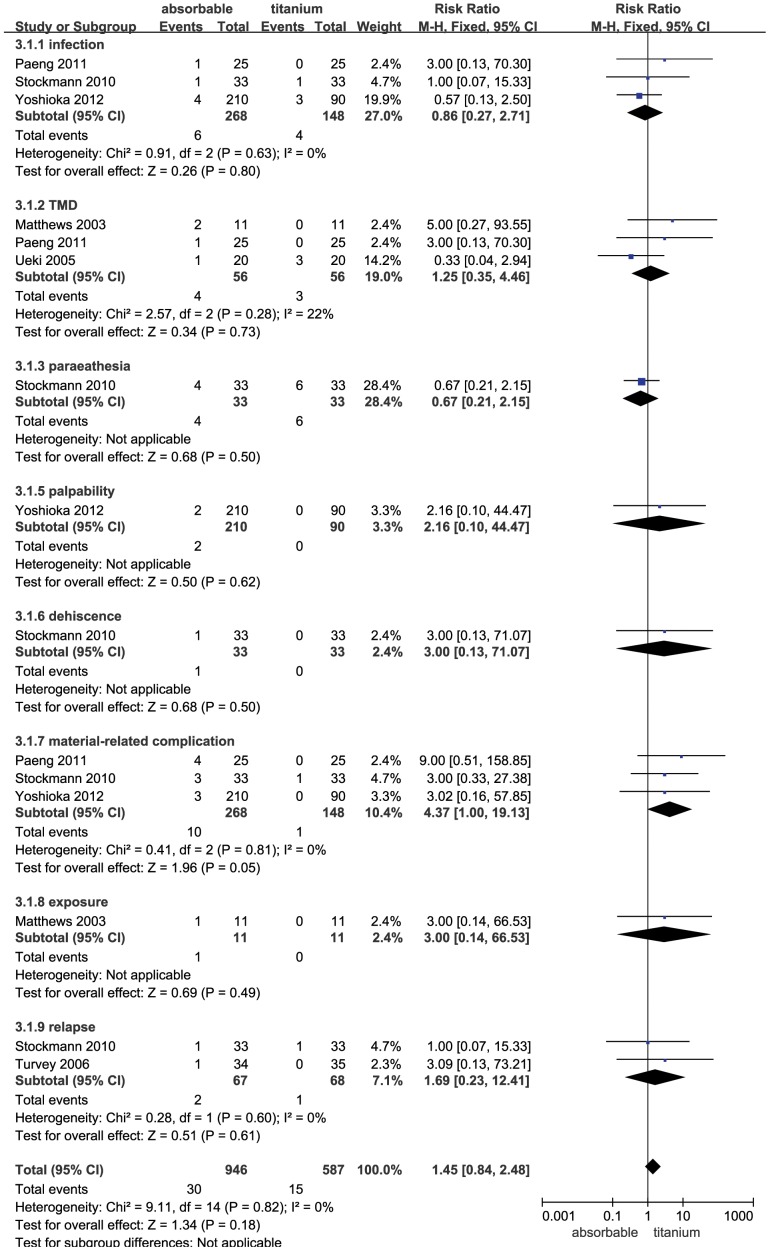
Forest plot of trials of absorbable fixation versus titanium examining the effect on relative risk of complications in BSSRO. See Figure 2 legend for expansion of abbreviations.

No significant difference was observed between the absorbable and titanium groups (RR = 1.45; 95% CI: 0.84–2.48; P = 0.18). The heterogeneity test was not substantial, as assessed by the I^2^ statistics (Q [d.f. = 14] = 9.11; P = 0.82; I^2^ = 0%) (Figure 5). A sub-group analysis was performed for complications, including infection, TMD, paraesthesia, palpability, dehiscence, material-related complications, exposure, and relapse. The results showed the same rate for these complications in the absorbable group and the titanium group (P>0.05). There was no evidence to suggest publication bias, as estimated by the funnel plot for the studies on complications in this operation (Figure 3c).

### Complication Comparison in the Le Fort I Operation

**Figure 6:**
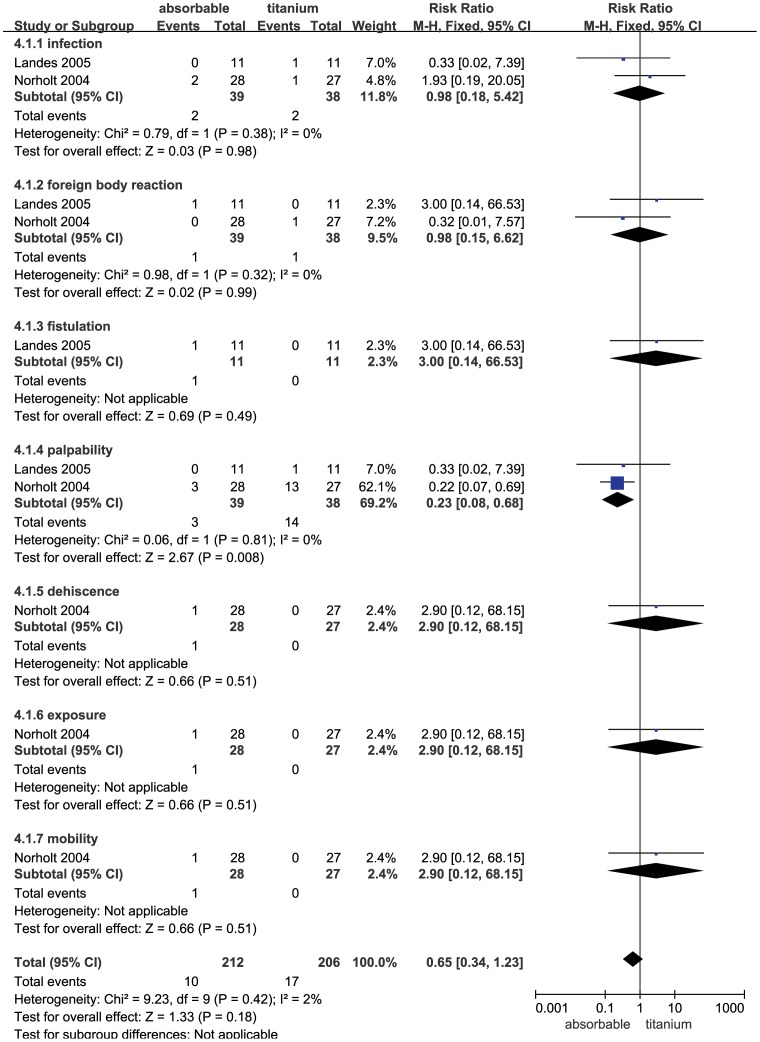
Forest plot of trials of absorbable fixation versus titanium examining the effect on relative risk of complications in Le Fort I. See Figure 2 legend for expansion of abbreviations.

There were only 2 trials comparing the complications of absorbable and non-absorbable fixation in the Le Fort I operation. There was no significant alteration observed between the absorbable and non-absorbable groups (RR = 0.65; 95% CI: 0.34–1.23; P = 0.18). The heterogeneity test was not substantial, as assessed by the I^2^ statistics (Q [d.f. = 9] = 9.23; P = 0.42; I^2^ = 2%) (Figure 6). A sub-group analysis was performed for complications, including infection, foreign body reaction, fistulation, palpability, dehiscence, exposure and mobility. Palpability (RR = 0.23; 95% CI: 0.08–0.68; P = 0.008) occurred significantly more frequently in patients fixed with titanium compared to patients receiving absorbable fixation. In addition, patients in the absorbable group were not associated with a more significant increase in infection (RR = 0.98; 95% CI: 0.18–5.42; P = 0.98), foreign body reaction (RR = 0.98; 95% CI: 0.15–6.62; P = 0.99), fistulation (RR = 3.00; 95% CI: 0.14–66.53; P = 0.49), dehiscence (RR = 2.90; 95% CI: 0.12–68.15; P = 0.51), exposure (RR = 2.90; 95% CI: 0.12–68.15; P = 0.51) and mobility (RR = 2.90; 95% CI: 0.12–68.15; P = 0.51). The funnel plot for the studies on complications in Le Fort I operation was relatively symmetrical, and publication bias was not evident (Figure 3d).

### Complication Comparison in the Fracture Fixation

**Figure 7:**
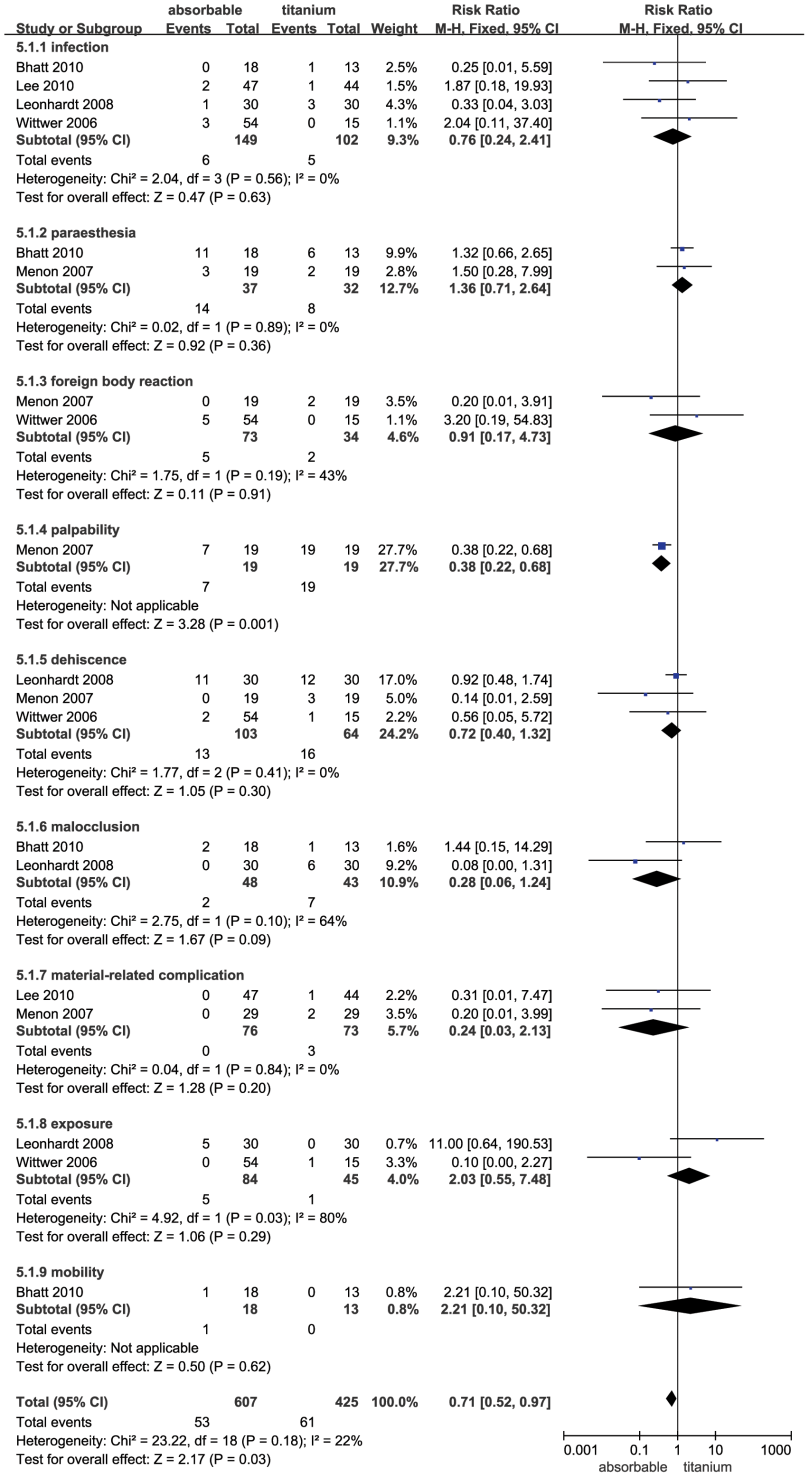
Forest plot of trials of absorbable fixation versus titanium examining the effect on relative risk of complications in fracture fixation. See Figure 2 legend for expansion of abbreviations.

Five trials were pooled in the fracture fixation operation. The combined results of the five trials revealed that the absorbable group had a significantly lower rate of complications compared to the titanium group (RR = 0.71; 95% CI: 0.52–0.97; P = 0.03). In addition, the heterogeneity was not observed (Q [d.f. = 18] = 23.22, P = 0.18, I^2^ = 22%) (Figure 7). A sub-group analysis was performed for complications, including infection, paraesthesia, foreign body reaction, palpability, dehiscence, malocclusion, material-related complication, exposure and mobility. Palpability (RR = 0.38; 95% CI: 0.22–0.68; P = 0.001) occurred significantly more frequently in patients fixed with titanium compared to patients receiving absorbable fixation. In addition, the absorbable group was not associated with a more significant increase in infection, paraesthesia, foreign body reaction, dehiscence, malocclusion, material-related complication, exposure and mobility (P>0.05). Publication bias was not evident, as estimated by the funnel plot for the studies on complications (Figure 3e).

## Discussion

To the best of our knowledge, this is the first meta-analysis to examine the safety profile of absorbable fixation system in maxillofacial surgery which can result in various complications. In addition to the common postoperative complications, including infection and sensory disturbance due to inferior alveolar nerve injury, TMD and relapse can occur [30], [31]. Recent developments have led to the introduction of titanium as a fixation material due to its superior qualities. In orthognathic surgery, bone fragments are usually fixed with the use of titanium plates and screws. However, the limitation of titanium fixation is the requirement of a subsequent removal operation, which is highly recommended. Although absorbable fixation systems avoid the need for a second operation, the potential complications that can occur, such as foreign body reaction, deter the wide use of absorbable fixation systems to be widely used. Thus, there is a need to systematically evaluate the safety of absorbable fixation systems in maxillofacial surgery.

Several clinical trials have shown the safety of absorbable fixation system in maxillofacial surgery. Observational studies in maxillofacial surgery have demonstrated that the bioresorbable plate leaves a stable bridge of healed bone or soft tissue after complete degradation with foreign-body reactions. Randomized, prospective controlled trials have shown no statistically significant differences in the incidence of material-related complications between the biodegradable and titanium groups [8], [32], [24]. However, Buijs et al. reported that biodegradable plates and screws performed inferiorly to titanium plates and screws in non-correct occlusion (11.1% vs. 8.8%), palpability plate/screw (50.4% vs. 38.1%), dehiscence (4.3% vs. 0%), abscess formation (9.4% vs. 3.5%) and inflammatory reactions (17.1% vs. 7.1%), respectively [1]. Most of the previous trials on absorbable fixation systems have systematically included patients receiving partial maxillofacial surgery. In this study, we found that absorbable fixation systems had a significantly higher rate of complications in maxillofacial surgery, especially with foreign body reaction and mobility. Foreign body reactions typically manifest with uniform histopathology, nonspecific inflammation, and abundant polymeric particles surrounded by mononuclear phagocytes and multinucleated foreign-body giant cells [33]. In addition, absorbable fixation systems cost more in clinical use. Therefore, absorbable fixation systems should not be considered as the first selective treatment materials for the management of bone fixation in maxillofacial surgery.

In maxillofacial surgery, the feasibility of applying biodegradable plates and screws for zygomatic fracture fixation was first demonstrated by Bos et al [34]. This technique soon extended to other craniomaxillofacial surgical procedures for fracture and orthognathic surgery. To explore the safety in different maxillofacial surgeries, four subgroups were described. In the bimaxillary operation categories, the application of an absorbable fixation system therapy did not have a significant increase in complications. There was no statistically significant difference in the foreign body reaction, relapse and mobility rates between fixation with titanium or absorbable plates/screws. Absorbable fixation of the single maxillary (Le Fort I) and single mandibular (BSSRO) seem to have a similar safety profile as titanium. A previous study also suggested the use of resorbable copolymer devices as a viable alternative to titanium for fixation of Le Fort I maxillary fixation [35]. In this study, fracture fixation included mandibular and zygomatic operation. Though they have similar stabilities, absorbable fixation is superior for fracture fixation, mainly in palpability. Palpability can be a problem for metallic fixation. In addition, long-term studies on the effects of metal osteosynthesis have identified the presence of metal ions in the vicinity of the site, leading to speculation that metal is gradually leached out by the action of body fluids [36]. Therefore, the application of an absorbable fixation system may be highly recommended for fracture fixation and an alternative option in the case of Le Fort I osteotomy, bimaxillary operation and BSSRO, compared to titanium.

There were several limitations of this study. First, the possibility of publication bias is always of concern. Although the enrolled trials consisted of more than 1500 patients in total, these results may be affected by publication bias. Second, heterogeneities between studies may confuse meta-analysis outcomes, such as with the use of different raw materials and source companies. Third, some of the trials were comparative studies, which are not as convincing. Therefore, more RCTs should be performed in order to obtain more convincing and reliable data to investigate the accuracy of this conclusion.

In conclusion, this meta-analysis found that absorbable fixation systems used for the fixation during maxillofacial surgery do not have adequate safety profiles. Notably, the occurrence of foreign body reactions and mobility were significantly more frequent in patients receiving absorbable fixation systems compared to titanium fixation. Subgroup indicated the safety of absorbable fixation systems was superior during fracture fixation. The absorbable fixation systems tend to have a similar favorable safety profile as titanium fixation during bimaxillary operation, BSSRO and Le Fort I operation, in which the absorbable fixation was superior to titanium fixation with regard to palpability. However, large-scale randomized, prospective trials of absorbable fixation systems used in maxillofacial surgery are needed, which will provide more convincing and reliable data regarding safety.

## Supporting Information

Text S1 Prisma Checklist.
(DOC)

Table S1 The basic characteristics of included studies.
(DOC)
