# Supplementary material for: Complications of Absorbable Fixation in Maxillofacial Surgery: A Meta-Analysis
Source: PLoS One. 2013 Jun 28;8(6):e67449. doi: 10.1371/journal.pone.0067449 (PMC3696084; doi:10.1371/journal.pone.0067449)
Supplement: Table S1 — The basic characteristics of included studies. (DOC) [file pone.0067449.s002.doc]

| Table S1. The basic characteristics of included studies | | | | | | | | | |
| --- | --- | --- | --- | --- | --- | --- | --- | --- | --- |
| author | year | study | disaese | operation | material | N | Age(yr) | FU(mo) | Jadad |
| Cheung | 2004 | RCT | dentofacial deformities | orth sur | resorbable | 30 | 22.9(16~37) | 24 | 4 |
|  |  |  |  |  | titanium | 30 |  | 24 |  |
| Landes | 2006 | CT | Ang II and III | Bi orth sur | P(L/DL)LA | 15 | 25(16~57) | 12 |  |
|  |  |  |  |  | titanium | 15 |  | 12 |  |
| Costa | 2006 | CT | Ang III | Bi orth sur | PLLA/PGA | 10 | -- | 12 |  |
|  |  |  |  |  | titanium | 12 | -- | 12 |  |
| Landes | 2007 | CT | Ang II and III | Bi orth sur | PLGA | 15 | 27(18~46) | 12 |  |
|  |  |  |  |  | titanium | 30 |  | 12 |  |
| Ahn | 2010 | CT | faci ammes | orth sur | resorbable | 120 | 23 | 12 |  |
|  |  |  |  |  | titanium | 152 |  | 12 |  |
| Tuovinen | 2010 | RCT | mandi/maxi growth disturb | Le Fort I  SSRO | PLDLA | 51 | 33.5 | 76 | 1 |
|  |  |  |  |  | titanium | 50 |  | 76 |  |
| Buijs | 2012 | RCT | mandi/maxi disturb/frac | Maxi sur | biodegradable | 117 | 31 (14-59) | 2 | 5 |
|  |  |  |  |  | titanium | 113 | 31 (16-60) | 2 |  |
| Matthews | 2003 | CT | Mandi retro | SSRO | SR-PLLA | 11 | 29(21~44) | 12 |  |
|  |  |  |  |  | titanium | 11 | 32(18~46) | 12 |  |
| Ueki | 2005 | RCT | mandi prog | SSRO | PLLA | 20 | -- | 12 | 3 |
|  |  |  |  |  | titanium | 20 | -- | 12 |  |
| Turvey | 2006 | CT | Mandi defi | SSRO | biodegradable | 34 | 27.5±13.0 | 12 |  |
|  |  |  |  |  | titanium | 35 | 26.8±11.2 | 12 |  |
| Stockmann | 2010 | RCT | Mandi jaw dis | SSRO | resorbable | 33 | 27±5.4 | 96 | 3 |
|  |  |  |  |  | titanium | 33 | 27±7.1 | 96 |  |
| Paeng | 2012 | CT | Ang III | SSRO | biodegradable | 25 | 22.6(18~30) | 6 |  |
|  |  |  |  |  | titanium | 25 | 25.3(18~33) | 6 |  |
| Yoshioka | 2012 | RT | Ang III | SSRO | biodegradable | 210 | 20(18~45) |  |  |
|  |  |  |  |  | titanium | 90 | 20(18~37) |  |  |
| Norholt | 2004 | RCT | Maxi dis | Le Fort I | PLLA/PGA | 28 | 23(17~48) | 12 | 2 |
|  |  |  |  |  | titanium | 27 | 22(17~50) | 12 |  |
| Landes | 2006 | CT | Maxi retro | Le Fort I | resorbable | 11 | 16~37 | 31,6~137 |  |
|  |  |  |  |  | titanium | 11 |  | 31,6~137 |  |
| Wittwer | 2006 | RT | zyg frac | Frac fix | LactoSorb | 18 | 34.6(17~71) | 24~44 |  |
|  |  |  |  |  | BioSorb | 18 |  |  |  |
|  |  |  |  |  | Delta | 18 |  |  |  |
|  |  |  |  |  | titanium | 15 | 35.5(18~69) |  |  |
| Menon | 2007 | CT | zyg frac | Frac fix | Delta | 19 | 30.5(20~41) | 12 |  |
|  |  |  |  |  | titanium | 19 | 31.3(21~51) | 12 |  |
| Leonhardt | 2008 | CT | Mandi frac | Frac fix | INION | 30 | 24(15~45) | 6 |  |
|  |  |  |  |  | titanium | 30 | 32(15~75) | 6 |  |
| Bhatt | 2010 | RCT | Mandi frac | Frac fix | bioresorbable | 18 | 26.6(18~46) | 2 | 5 |
|  |  |  |  |  | titanium | 13 | 28.7(18~48) | 2 |  |
| Lee | 2010 | CT | Mandi frac | Frac fix | P(L/DL)LA | 47 | 11~69 | 12 |  |
|  |  |  |  |  | titanium | 44 |  | 12 |  |

FU = follow up; Mo = months; RCT = randomized controlled trial; CT = comparative trial; RT = randomized trial; yr = year; SSRO = sagittal split ramus osteotomy; N = number; orth sur = orthognathic surgery; Ang II and III = Angle class II and III dysgnathia; Bi orth sur = bimaxillary orthognathic surgery; faci ammes = facial ammestry; Maxi sur = maxillofacial surgery; Mandi defi = mandibular deﬁciency; Mandi jaw dis = mandibular jaw disproportion; Maxi dis = maxillary growth disturbances; Maxi retro = maxillary retrognathia; zyg frac = zygoma fracture; Frac fix = fractur fixation.
